# MicroRNAs in thyroid eye disease: expression signatures and clinical implications

**DOI:** 10.3389/fimmu.2026.1766372

**Published:** 2026-03-23

**Authors:** Xinyu Wang, Hongyue Chen, Songwei Li

**Affiliations:** 1 The Department of Thyroid and Breast Surgery, The Second Affiliated Hospital of Henan University of Chinese Medicine, Zhengzhou, China; 2 The Department of Rheumatology ,The First Affiliated Hospital of Henan University of Chinese Medicine, Zhengzhou, China

**Keywords:** GO, miRNA, pathogenic mechanism, TAO, TED, therapeutic prospects

## Abstract

Thyroid eye disease (TED), also known as Graves’ ophthalmopathy (GO) or thyroid-associated ophthalmopathy (TAO), is an autoimmune orbital disease. The core pathogenic mechanism is immune dysregulation triggered by self-antigens, which activates orbital fibroblasts and leads to pathological changes such as tissue hyperplasia and fibrosis. The clinical manifestations include proptosis and eyelid retraction. MicroRNA (miRNA), as a key factor in post-transcriptional regulation, is widely involved in the occurrence and development of TED. Altered expression profiles of specific miRNAs, such as miR-21, miR-146a, and miR-144-3p, have been observed in clinical samples and *in vitro* fibroblast models, where they are associated with processes including fibroblast activation, fibrosis, adipogenesis, and inflammation. These findings highlight miRNAs as potential mechanistic targets for further investigation. This review summarizes the pathogenesis of TED and discusses the emerging role of miRNAs based on current clinical and experimental evidence, aiming to provide insights for future research and therapeutic development.

## 1 Introduction

Thyroid eye disease (TED), also known as Graves’ ophthalmopathy (GO) or thyroid-associated ophthalmopathy (TAO), is an autoimmune orbital disorder frequently associated with thyroid dysfunction. Its pathogenesis is characterized by inflammation, fibrosis, and adipogenesis within the orbit. Patients commonly present with a spectrum of symptoms including proptosis, eyelid retraction, restricted ocular motility, and ocular surface discomfort. In severe cases, the disease can progress to compressive optic neuropathy (1). For consistency, the term ‘TED’ will be used throughout this manuscript. TED imposes a considerable global disease burden, with an estimated incidence of 0.5%. It demonstrates a strong female predominance and an incidence peak among individuals aged 30–50 years (2). The etiology of TED involves a complex interplay of genetic, immune, and environmental factors. A central mechanistic pathway is the interaction between the thyroid-stimulating hormone receptor (TSHR) and the insulin-like growth factor-1 receptor (IGF-1R), which drives orbital inflammation and tissue remodeling, ultimately leading to characteristic facial changes and visual impairment (3). Although most cases of TED are mild or self-limiting, approximately 30% of patients require anti-inflammatory or immunosuppressive therapy. Current treatment options, however, often yield unsatisfactory outcomes (4). Glucocorticoids, the first-line treatment drugs, are hampered by suboptimal response rates, significant side effects, and a high risk of relapse upon discontinuation. Other interventions, such as radiotherapy, immunosuppressive agents, and orbital decompression surgery, are frequently limited by variable efficacy, inherent invasiveness, and restricted applicability (5). Therefore, a deeper understanding of TED pathogenesis is essential for developing targeted therapies to address this pressing clinical challenge.

## 2 Overview of miRNA

### 2.1 Introduction to miRNA

MicroRNAs (miRNAs) are a class of evolutionarily conserved, single-stranded non-coding RNAs approximately 19–25 nucleotides in length. They function as key post-transcriptional regulators of gene expression, primarily by binding to the 3’ untranslated region (3’ UTR) of target mRNAs, leading to mRNA degradation or translational repression (6, 7). It is estimated that miRNAs may regulate more than 30% of human genes, underscoring their broad influence on diverse biological processes, including development, cell proliferation, differentiation, apoptosis, and metabolism (8, 9). The biogenesis of miRNAs is a multi-step process. It begins with the transcription of primary transcripts (pri-miRNAs) in the nucleus, which subsequently undergo a series of enzymatic processing steps to yield mature, functional miRNAs in the cytoplas (6). Since their discovery in the early 1990s, miRNAs have been established as critical modulators of a wide array of immune and cellular functions (10).

### 2.2 miRNA biological occurrence

The biogenesis of miRNAs is a multi-step, tightly regulated process. It begins in the nucleus with RNA polymerase II-mediated transcription of miRNA genes, yielding primary miRNA transcripts (pri-miRNAs) that contain a 5’ cap and a 3’ poly(A) tail and harbor one or more stem-loop structure (11). The microprocessor complex, comprising Drosha and DGCR8, recognizes and cleaves the pri-miRNA to release a ~70-nucleotide precursor miRNA (pre-miRNA) (12). This step is finely regulated by various factors; for instance, the RNA helicase Brr2a interacts with HYL1 to facilitate pri-miRNA structural remodeling, thereby promoting miRNA maturation (13). The pre-miRNA is then exported to the cytoplasm via exportin-5 in a Ran-GTP-dependent manne (14, 15), where it is cleaved by the RNase III enzyme Dicer to generate the mature miRNA duplex (16).

Notably, a subset of miRNAs can be processed independently of Dicer, through direct cleavage mediated by Argonaute 2 (Ago2) (17). The mature miRNA is subsequently loaded into the RNA-induced silencing complex (RISC), guiding the miRISC to target mRNAs via complementary base pairing with the 3’ untranslated region (3’ UTR). This interaction leads to translational repression or mRNA degradation, enabling post-transcriptional gene regulation (18, 19). Multiple layers of control govern miRNA biogenesis and function. At the transcriptional level, transcription factors and epigenetic modifications collectively modulate miRNA gene expression (20). During processing, the activities of Drosha and Dicer are regulated through dynamic protein interactions and post-translational modifications. Additionally, RNA-binding proteins such as TAR DNA-binding protein-43 (TDP-43) can integrate into the microprocessor to enhance maturation efficiency (21). Furthermore, epigenetic mechanisms—such as methylation of miRNA gene promoters or histone acetylation—can modulate the transcriptional expression levels of miRNAs, thereby exerting an additional layer of regulation on their downstream functional networks (22). In summary, miRNA biogenesis—from transcription to functional RISC assembly—comprises multiple rigorously ordered steps, each subject to precise spatiotemporal control. This elaborate, multi-tiered regulatory network ensures the fidelity and efficacy of miRNA-mediated gene silencing.

### 2.3 Overview of miRNA functions

MicroRNAs (miRNAs) are a class of endogenous short non-coding RNAs that function as key post-transcriptional regulators of gene expression, primarily through promoting mRNA degradation or translational inhibition (19). It is estimated that nearly 3,000 miRNAs exist in the human genome. A defining feature of miRNAs is their ability to simultaneously target numerous mRNAs; a single miRNA can typically regulate hundreds of distinct transcripts, thereby serving as a molecular hub that coordinates entire gene networks and modulates complex biological pathways (23, 24). As well-established regulators of core cellular processes—including proliferation, apoptosis, differentiation, and metabolism (25). miRNAs play vital roles in diverse physiological and pathological contexts. Furthermore, emerging epigenetic evidence highlights miRNAs as critical mediators of cell proliferation, where they orchestrate sophisticated gene expression programs that ultimately determine cellular outcomes (26).

MicroRNAs(miRNAs) serve as central regulators at the immunological level, modulating essential processes such as immune cell development, differentiation, antibody production, and inflammatory responses. Their critical role is exemplified by their ability to ensure coordinated crosstalk between innate and adaptive immunity (27). As key modulators of immune function, miRNAs influence T-cell reactivity, antibody production, and the maintenance of chronic inflammation (28). Dysregulation of miRNA expression can disrupt immune homeostasis and self-tolerance, thereby contributing to the pathogenesis of autoimmune diseases (29). The dysregulation of miRNA expression is strongly associated with the development of various significant human diseases, including tumors, diabetes, Alzheimer’s disease, and autoimmune disorders, highlighting their potential role in disease pathogenesis (30). This characteristic also makes miRNAs an important source for disease diagnostic markers and potential therapeutic targets.

## 3 The mechanism of miRNA in regulating TED and its future therapeutic prospects

TED is a chronic autoimmune disorder characterized by selective targeting of orbital tissues. The disease is initiated by a breakdown in immune tolerance, with pathological activation and differentiation of orbital fibroblasts (OFs) representing a central cellular event. These processes collectively drive tissue remodeling, functional impairment, and the characteristic clinical manifestations of TED.

**Figure 1:**
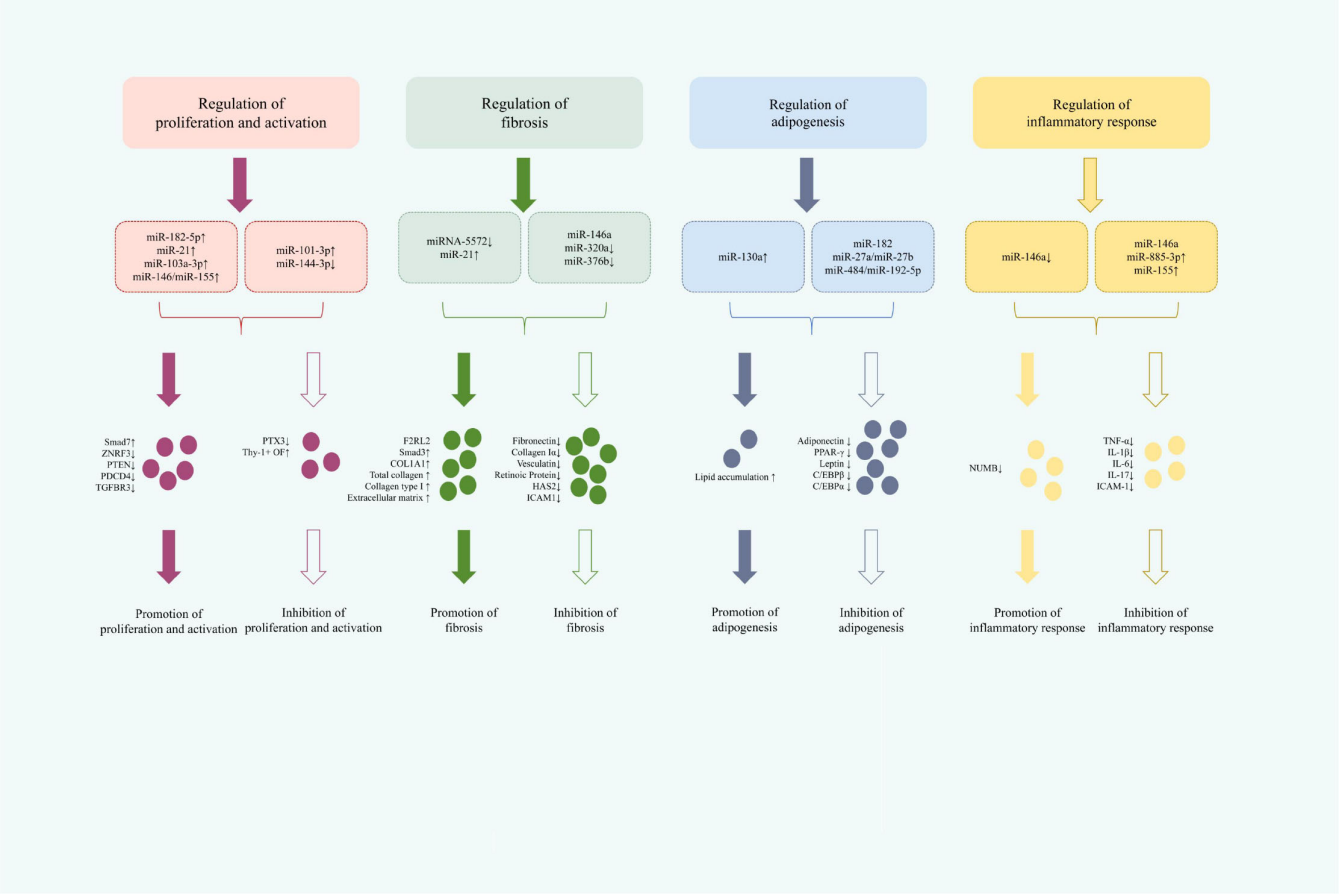
Summary of the mechanism of miRNA treatment for TED.

In recent years, miRNAs have emerged as critical regulators within the pathogenic network of TED. As key post-transcriptional modulators, they significantly contribute to disease pathogenesis and represent promising targets for therapeutic intervention. At the molecular level, miRNAs regulate multiple pathological processes, including of proliferation and activation, fibrotic remodeling, adipogenic differentiation, and inflammatory signaling. Elucidating these diverse mechanisms provides multifaceted insights and opens new avenues for the targeted treatment of TED. (**Figure 1** summarizes the mechanism of miRNA in treating TED).

### 3.1 The role of miRNAs in OFs proliferation and activation

Orbital fibroblasts (OFs) are central effector cells in the pathogenesis of TED (31). A defining characteristic of OFs is their specific expression of IGF-1R and TSHR, which mediate cytokine secretion, regulate adipogenesis, and drive connective tissue remodeling, thereby critically influencing TED progression (32). As tissue-resident sentinels, OFs become abnormally activated and secrete large quantities of inflammatory mediators, facilitating persistent immune cell recruitment into the orbit. This leads to reciprocal interactions between OFs and immune cells, forming a pro-inflammatory feedback loop that sustains and amplifies orbital inflammation (33). Furthermore, OFs exhibit a remarkable capacity for pathological tissue remodeling. Their aberrant proliferation and differentiation into adipocytes or myofibroblasts represent key mechanisms underlying the increase in orbital tissue volume and the development of fibrotic lesions (34, 35). Activated OFs not only display enhanced proliferative activity but also actively synthesize extracellular matrix components—particularly hyaluronic acid—collectively driving tissue expansion, structural remodeling, and fibrosis in TED (36).

Collectively, this review underscores that the proliferation and activation of orbital fibroblasts (OFs) constitute a fundamental pathological process in TED. Acting as principal cellular effectors, OFs mediate the conversion of systemic immune signals into localized orbital pathology. Targeting the key mechanisms driving OFs activation therefore represents a promising and strategic frontier for advancing TED therapy.

Functional studies have demonstrated that specific miRNAs distinctly regulate OFs proliferation and activation. For instance, the upregulation of miR-182-5p, miR-21, miR-103a-3p, miR-146a, and miR-155 promotes proliferation, whereas elevated levels of miR-101-3p or knockdown of miR-144-3p exert suppressive effects.

**Figure 2:**
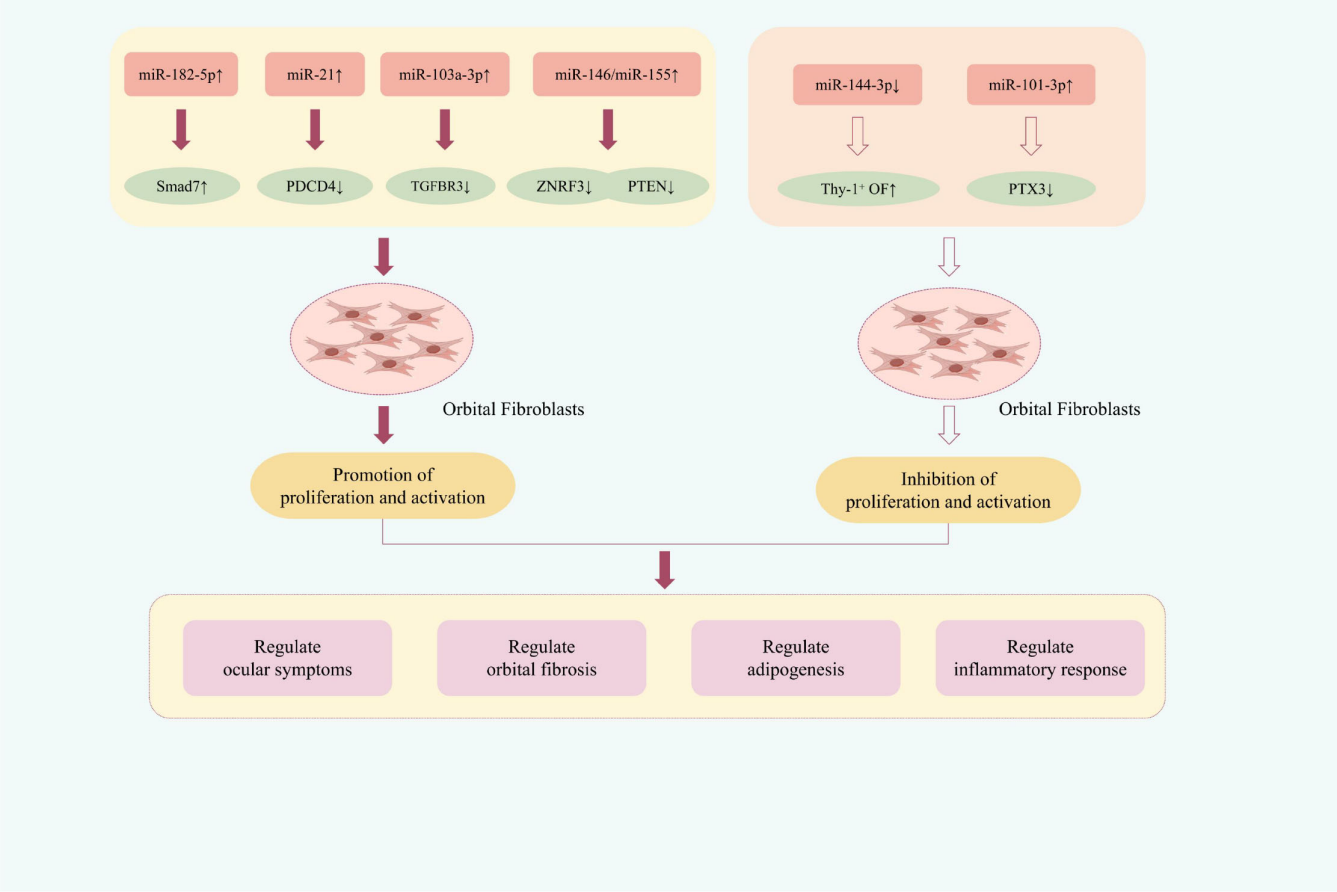
Mechanisms of miRNA-regulated the proliferation and activation of OFs in TED therapy.

In the lacrimal glands of patients with Thyroid Eye Disease (TED), populations of CD34^+^ and CD34^−^ cells with fibroblast-like characteristics are expanded. Under the influence of cytokines such as IL-17A and IL-6, CD34^+^ OFs can differentiate into myofibroblasts or adipocytes (37, 38). The expression of miR-182-5p is significantly upregulated in the orbital connective tissues orbital connective tissue of TED patients. Mechanistically, miR-182-5p promotes proliferation and suppresses apoptosis in CD34^+^ OFs by directly targeting Smad7, collectively enhancing their wound repair capacity and implicating it in TED pathogenesis (39). miR-101-3p, known to regulate proliferation, apoptosis, and fibrosis in various diseases (40, 41), is significantly dysregulated in peripheral blood mononuclear cells from TED patients (42). Pentraxin 3 (PTX3) is a typical acute-phase protein that promotes cell proliferation. Its overexpression can stimulate the proliferation and differentiation of human osteoblasts (43). The study found that the expression of miR-101-3p was significantly downregulated in TED-OFs and orbital adipose tissue. Upregulation of miR-101-3p expression could downregulate the expression of pentraxin 3 (PTX3), significantly reduce the survival ability of TED-OFs, inhibit the proliferation of TED-OFs, and ultimately delay the progression of TED (44). Platelet-derived growth factor (PDGF) promotes myofibroblast proliferation and survival, contributing to tissue repair and fibrosis (45). Programmed cell death 4 (PDCD4) is a tumor suppressor gene related to the cell cycle and apoptosis, and has become an important functional target of miRNA-21 (46). The research found that PDGF-BB can upregulate the expression of miRNA-21, inhibit the expression of PDCD4, significantly promote the proliferation of OFs, and thereby facilitate the formation of TED (47). Thus, inhibiting miRNA-21 may hold therapeutic value in TED. In various organ and tissue fibrosis conditions, transforming growth factor β1 (TGFβ1) is closely related to the activation of myofibroblasts and the synthesis of extracellular matrix (ECM) (48). Its elevated expression in TED-OFs and ocular soft tissues underscores its role in orbital fibrosis (49). By collecting and analyzing the miRNA expression profiles of TED and normal track samples, the differentially expressed miR-103a-3p was identified. Subsequent experiments demonstrated that upregulating the expression of miR-103a-3p could inhibit the expression of transforming growth factor β receptor 3 (TGFBR3), and through the Erk-JNK and TGF-β1/SMAD signaling pathways, significantly enhance the activity of OFs; and increase the expression of vimentin, fibronectin, and fibronectin-associated proteins, promoting the activation of TED-OFs and fibrosis (50). Therefore, inhibiting miR-103a-3p may therefore mitigate fibrosis in TED. The thyroid stimulating hormone receptor (TSHR) is a G protein-coupled receptor (GPCR) that induces the production of cAMP and the PI3K/Akt signaling cascade, which can promote the production of hyaluronic acid and fat formation in the OFs of TED patients (51). In TED, the expressions of miR-146a and miR-155 were significantly upregulated. Both of them are involved in regulating cell proliferation, survival and inflammatory responses; their expression balance has a crucial impact on disease progression, when miR-155 is dominant, it exacerbates inflammation and autoimmunity, while when miR-146a is dominant, it may alleviate the pathological process (52). ZNRF3 and PTEN, as negative regulators of cell proliferation, are respectively the target genes of miR-146a and miR-155 (53, 54). Further studies have shown that activation of TSHR can upregulate the expression of miR-146a and miR-155, thereby inhibiting ZNRF3 and PTEN, and promoting the proliferation and fibrosis OFs (55). This suggests that targeting the inhibition of miR-146a/155 expression may become a potential therapeutic strategy for intervening in the process of TED. Further evidence implicates miRNA dysregulation in TED pathogenesis, as investigators reported that coordinated upregulation of miR-155 and downregulation of miR-146a significantly induced orbital fibroblast proliferation. This miRNA profile was further shown to stimulate the expression of inflammatory mediators (TNF-α, IL-6, IL-8) and key fibrotic components (collagen I, hyaluronic acid, MMP-9), thereby promoting both ocular inflammation and tissue fibrosis (56). Exosomes(Exos) are extracellular vesicles with a diameter of approximately 40–160 nanometers, serving as a crucial carrier for intercellular communication (57). Plasma exosomes (Pla-Exos) and the miRNAs they carry can regulate immune activation, and play a significant role in TED and other autoimmune diseases by influencing inflammation, fibrosis, and cell proliferation/apoptosis processes (58, 59). miR-144-3p, as a miRNA derived from specific immune cells, is significantly upregulated in peripheral blood mononuclear cells (PBMCs) of TED patients. miR-144-3p can reduce the viability and proliferation ability of Thy-1+ OFs; increase the expression of IL-1β, IL-6, TNF-α, etc., promoting the inflammatory response; and enhance the expression of hyaluronic acid (HA), driving the fibrotic process (60). (**Figure 2** illustrates miRNA-regulated the proliferation and activation of OFs in TED therapy).

### 3.2 The role of miRNAs in fibrosis

Fibrosis is a pathological process of abnormal tissue proliferation and scarring due to the excessive accumulation of extracellular matrix (ECM) components, most notably collagen, which leads to tissue hardening and loss of function (61). Myofibroblasts serve as the central drivers of fibrosis, directly mediating the pathological deposition and structural remodeling of the ECM (62). In the progression of TED, remodeling and fibrosis of orbital soft tissues gradually emerge as prominent pathological hallmarks (63). During this process, transforming growth factor-β (TGF-β) acts as a key pro-fibrotic mediator that activates fibroblasts and stimulates the synthesis of various fibrotic molecules, including type I and IV collagen, connective tissue growth factor (CTGF), and fibronectin, thereby exacerbating pathological ECM accumulation (64). As TED advances, fibrosis becomes the dominant pathology, resulting in structural deformities and functional deficits that are often irreversible. At this stage, therapeutic strategies shift from medical to surgical management, as immunosuppressive agents show limited efficacy. Interventions such as orbital decompression or extraocular muscle surgery are often required to correct disfigurement and restore functional vision (65). Fibrosis represents the end stage of TED progression and serves as a key pathological indicator of poor prognosis. Therefore, early intervention and prevention of fibrosis during the disease course are critical for preserving patients’ visual function.

At the molecular level, multiple miRNAs have been identified as key regulators of the fibrotic process. For instance, downregulation of miR-5572 and upregulation of miR-21 exert pro-fibrotic effects, whereas downregulation of miR-146a, miR-320a, and miR-376b can effectively attenuate fibrosis development.

**Figure 3:**
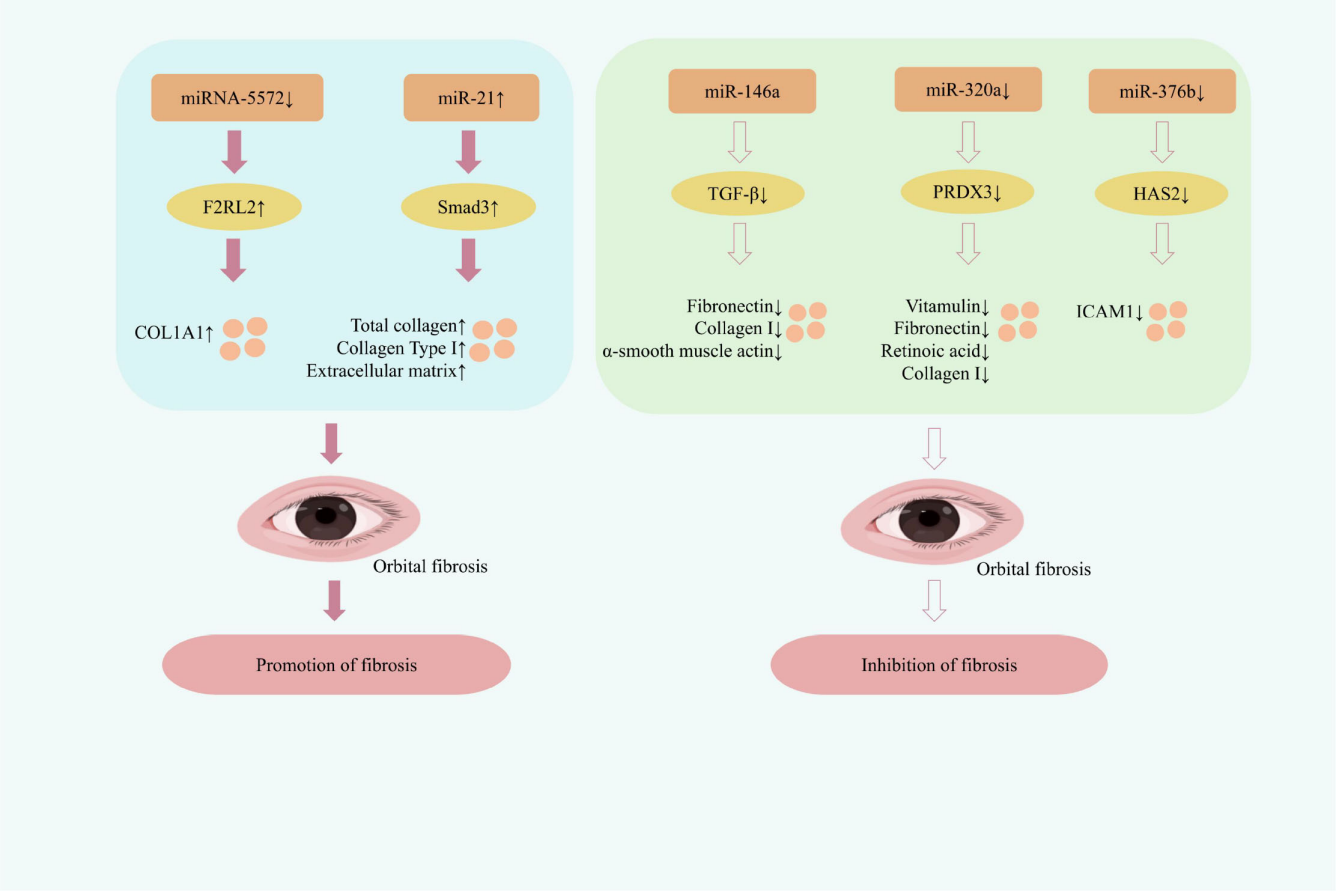
Mechanisms of miRNA-mediated regulation of fibrosis in TED therapy.

The thrombin receptor-like 2 (F2RL2), also known as protease-activated receptor 3 (PAR-3), mediates the effects of thrombin on diverse cellular processes including proliferation, migration, inflammation, growth factor release, and extracellular matrix synthesis (66). Elevated expression of PAR-3 has been observed in the lungs of patients with idiopathic pulmonary fibrosis, where it enhances thrombin-induced epithelial-mesenchymal transition (EMT), thereby promoting fibrotic development (67). In TED, downregulation of miR-5572 directly targets F2RL2/PAR-3, leading to increased expression of collagen type I alpha 1 chain (COL1A1) in OFs and contributing to fibrosis (68). Conversely, miR-146a has been identified as a suppressor of TGF-β-driven fibrosis, downregulating key fibrotic components such as fibronectin, collagen Iα, and α-smooth muscle actin, effectively inhibiting fibrosis progression (69). miR-320a is implicated in promoting fibrosis in various contexts, including cardiovascular diseases, where it activates the PI3K/Akt/mTOR signaling pathway to stimulate cardiac fibroblast proliferation (70). Additionally, miR-320a inhibits collagen degradation in chondrocytes and modulates MMP-13 expression, further exerting pro-fibrotic effects (71). Some researchers have identified differentially expressed miRNAs in TED that may be related to fibrosis. They have identified miR-320a in clinical samples and in OFs treated with transforming growth factor β1 (TGF-β1), and determined that miR-320a is a potential regulatory factor in the pathogenic mechanism of TED. PRDX3 is the downstream target of miR-320a. By inhibiting the expression of miR-320a, PRDX3 expression can be downregulated, and the expressions of vitronectin, fibronectin, Retinoic acid receptor alpha (RARα) and collagen I can be reduced, thereby reducing fibroblast activation and fibrosis changes, and improving the pathological damage of TED mice. In addition, this intervention was shown to mitigate oxidative stress by reducing the levels of ROS and Malondialdehyde(MDA), while simultaneously enhancing the activity of SOD and the content of GSH. These changes corresponded with significant alleviation of ocular inflammation and improved orbital mobility scores in TED mice (72). Therefore, reducing the expression of miR-320a and targeting the miR-320a/PRDX3 axis is of great significance for alleviating oxidative stress and fibrotic changes in TED. Hyaluronan synthase 2 (HAS2) catalyzes the production of hyaluronic acid (HA), a major non-sulfated glycosaminoglycan. Accumulation of HA in orbital tissues contributes to tissue expansion and edema in TED (73). In this context, HAS2-derived HA engages receptors such as CD44, TLR2, and TLR4, exacerbating local inflammation and tissue swelling (74). Nevertheless, the precise role and regulation of this pathway in TED pathogenesis require further validation through studies specifically focused on TED-derived orbital tissues or relevant cellular models. In patients with active TED, the expression of miR-376b is significantly reduced. It can inhibit fibrosis by targeting and suppressing the expression of hyaluronic acid synthase 2 (HAS2) and intercellular adhesion molecule 1 (ICAM1); and it can also down-regulate the expression of TNF-α, IL-6, etc., exerting anti-inflammatory effects (42). Among regulators of fibrosis, miR-21 is a well-characterized multifunctional miRNA involved in various physiological and pathological processes, including cancer, stem cell biology, and aging (75). TGF-β drives fibrosis partly by modulating specific miRNAs, such as repressing miR-29 and inducing miR-192 to promote collagen deposition. Moreover, TGF-β consistently upregulates miR-21, a central mediator of fibroblast activation and fibrosis in multiple organs including the lungs and kidneys (76, 77). A central mechanism in TED fibrosis involves the upregulation of miR-21 in orbital fibroblasts. *In vitro*, miR-21 overexpression activates the TGF-β1/SMAD pathway by enhancing Smad3 phosphorylation, thereby coordinating multiple pro-fibrotic events. These include promoting proliferation (via Ki67), inhibiting apoptosis (via Bcl-2/Bax), driving myofibroblast differentiation (via α-SMA), and accelerating ECM accumulation (via collagen I), which collectively exacerbate the fibrotic pathology (78). Therefore, reducing miR-21 expression may help alleviate inflammation, suppress abnormal OFs proliferation, and attenuate fibrosis. Developing inhibitors targeting miR-21 is thus considered a promising therapeutic strategy for intervening in TED-associated fibrosis. (**Figure 3** depicts miRNA-regulated fibrosis mechanism for TED treatment).

### 3.3 The role of miRNAs in adipogenesis

Adipogenesis constitutes the core pathological process underlying the structural lesions in TED, which interacts with inflammatory and fibrotic pathways to form a vicious cycle (79). This process is primarily characterized by the pathological proliferation and expansion of adipose tissue within the orbit. The fundamental mechanism involves the abnormal differentiation of orbital precursor adipocytes and the consequent increase in adipose volume (80). In TED, the differentiation of activated orbital fibroblasts into adipocytes represents a critical step, directly leading to typical clinical manifestations such as proptosis and periorbital swelling (81). Notably, activated OFs exhibit bidirectional differentiation potential: they can differentiate into fibrosis-promoting myofibroblasts or into adipocytes that contribute to pathological fat accumulation (82). The expression level of Thy1 (CD90) is a key factor determining the differentiation fate of OFs: orbital fibroblasts with high Thy1 expression (Thy1^+^) tend to differentiate into myofibroblasts, whereas those with low or negative Thy1 expression (Thy1^−^) predominantly undergo adipogenic differentiation (35). Given the central role of adipogenesis in TED pathogenesis, targeted inhibition of this process holds significant clinical potential for ameliorating ocular symptoms in affected patients.

Through summarization, it was found that at the molecular regulation level, different miRNAs play antagonistic roles: the upregulation of miR-130a has a positive regulatory effect, promoting adipogenesis; while miR-182, miR-27a/miR-27b, and miR-484/miR-192-5p exert negative regulatory effects, effectively inhibiting adipogenesis.

**Figure 4:**
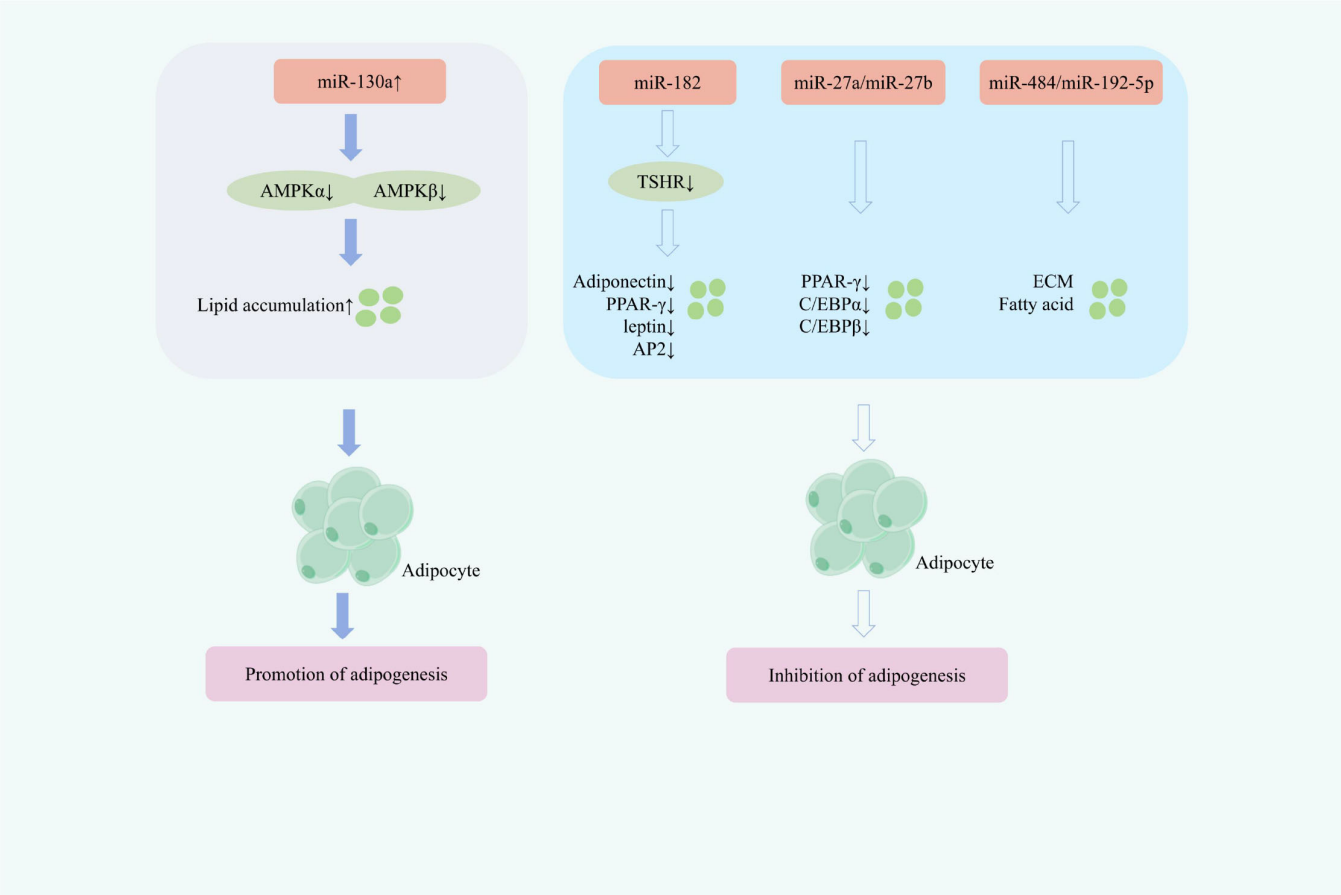
Mechanisms of miRNA-regulated adipogenesis for TED treatment.

In thyroid eye disease (TED), OFs with low expression of thymocyte differentiation antigen-1 (Thy-1) exhibit a higher adipogenic potential and a distinct miRNA profile, among which miR-130a is notably upregulated (35). miR-130a contributes to orbital tissue remodeling in TED by promoting adipogenesis and lipid accumulation (83). A study found that the expression of miR-130a was significantly upregulated in Thy1-OFs. Subsequent research results indicated that increasing the expression of miR-130a could reduce the expression of AMPKα and AMPKβ, inhibit the activation of AMPK, and increase lipid accumulation in OFs, thereby leading to excessive accumulation of orbital adipose tissue and promoting fatogenesis (84). This mechanism suggests that targeted inhibition of miR-130a holds great potential for treating the adipose hyperplasia lesions in TED. E74-like factor 3 (ELF3), a key member of the E-26 (ETS) transcription factor family, can target the promoter of miR-182 and positively regulate its expression, participating in the pathophysiology of various immune-related diseases (85). Previous research has reported a significant increase in miR-182 expression alongside decreased thyroid-stimulating hormone receptor (TSHR) levels in patients with TED and in 3T3-L1 adipocytes. Further studies demonstrate that miR-182 inhibits TSHR expression and downregulates adiponectin, leptin, PPAR-γ, and AP2, ultimately suppressing adipogenesis in TED (86). Another study observed marked downregulation of miR-27a and miR-27b in orbital tissues from TED patients compared with healthy controls (87). Subsequent investigations revealed that miR-27a/miR-27b reduce the expression of peroxisome proliferator-activated receptor γ (PPARγ), CCAAT/enhancer-binding protein α (C/EBPα), and C/EBPβ, significantly decreasing Oil Red O-stained lipid droplet content and thereby inhibiting adipogenesis (88). A comparative analysis of miRNA profiles between active and inactive TED patients identified 10 differentially expressed miRNAs. Functional enrichment analysis of their target genes highlighted significant involvement in fatty acid metabolism and extracellular matrix (ECM) receptor interactions. Notably, circulating levels of miR-484 and miR-192-5p were significantly lower in active TED patients than in those with inactive disease, suggesting their potential utility as circulating biomarkers for assessing TED disease activity (89). (**Figure 4** demonstrates miRNA-regulated Adipogenesis in TED therapy).

### 3.4 The role of miRNAs in inflammatory response

The orbital inflammatory response represents the core pathological process in TED. In the acute phase, this response is primarily driven by a Th1 cell-mediated immune reaction; as the disease progresses, the chronic inactive phase is gradually dominated by fibrotic changes (90). The inflammatory response not only serves as a key driver of TED onset and progression (91), but also further promotes adipogenesis and fibrosis, directly contributing to typical clinical manifestations such as proptosis, tissue edema, and pain (92). As a key mediator in the inflammatory process, cytokines play a bridging role in TED. Their changes are significantly correlated with ocular surface lesion parameters and the overall disease activity (93). Activated T cells and B cells infiltrate the orbital connective tissue, releasing cytokines including IL-1β, IL-6, TNF-α, TGF-β1, as well as autoantibodies such as IgG, ultimately resulting in tissue inflammation, fibrosis, and structural remodeling (94). Therefore, effective suppression of the inflammatory response in the early stages of the disease is essential for halting disease progression and preventing irreversible damage.

Through the summary of current research, it is found that the down-regulation of miR-146a will exacerbate inflammation; while the up-regulation of miR-146a, miR-885-3p, and miR-155 can all inhibit the inflammatory response.

**Figure 5:**
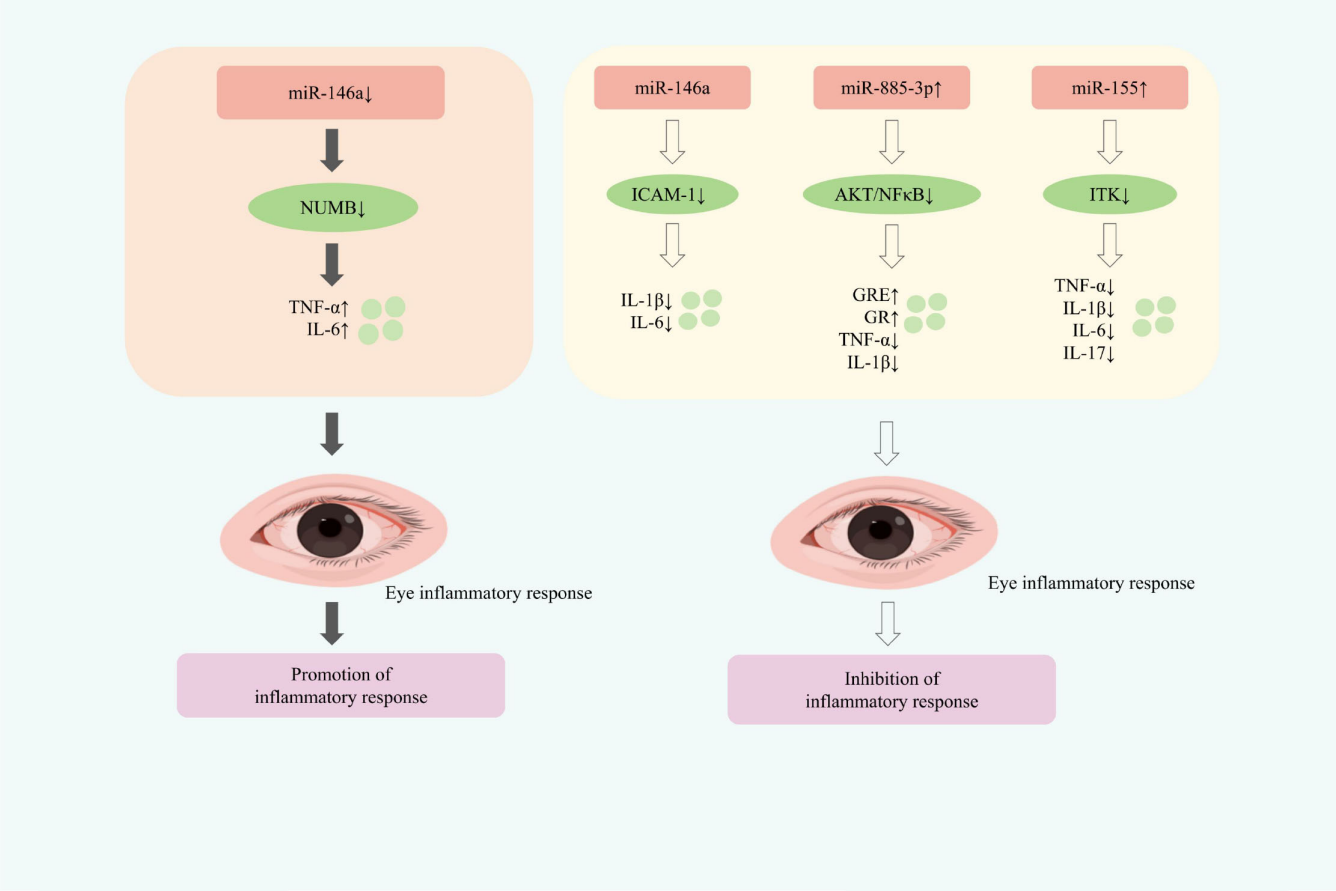
Mechanisms of miRNA regulation of inflammatory responses in TED therapy.

NUMB is a key regulatory gene involved in cell proliferation and differentiation (95). As an important target of miR-146a, NUMB activity is modulated by this microRNA. Studies have shown that miR-146a is downregulated in CD4^+^ T cells of patients with active TED, and this downregulation inhibits NUMB function, thereby promoting orbital inflammation (96). These findings suggest that restoring miR-146a expression may represent a potential therapeutic strategy for mitigating inflammation in TED. Consistently, miR-146a expression is significantly higher in TED orbital adipose tissue compared with non-TED controls. Functionally, miR-146a reduces the expression of IL-6 and ICAM-1 proteins induced by IL-1β, attenuating the inflammatory response in the orbital region (97). Intravenous glucocorticoids (IVGCs) remain the first-line treatment for moderate to severe active TED (98). In patients responsive to IVGCs, plasma exosomal miR-885-3p levels are elevated. Exosomes derived from clinically improved patients (SI-exo) can upregulate miR-885-3p expression, inhibit the AKT/NF-κB signaling pathway, enhance glucocorticoid response element (GRE) activity and glucocorticoid receptor (GR) expression, and downregulate inflammatory factors in OFs, thereby significantly improving glucocorticoid sensitivity (99). miR-155 is considered a key driver in the pathogenesis of TED (100). It can inhibit key negative regulatory factors such as cytokine signal transduction inhibitor 1 (SOCS1) and inositol-5-phosphatase 1 with SH2 domain (SHIP1), thereby promoting the inflammatory response (101, 102). While miR-146a can inhibit T-cell activation and modulate immune responses (103). Both miRNAs are also involved in cell proliferation, differentiation, and other essential processes (104). IL-2-inducible T-cell kinase (ITK), a non-receptor tyrosine kinase expressed in T cells, NK cells, and mast cells, plays a central role in T-cell receptor and Fc receptor signaling and is implicated in autoimmune pathogenesis (105). ITK contains a binding site for miR-155 and is a direct target of this miRNA. Upregulation of miR-155 suppresses ITK expression and reduces levels of TNF-α, IL-1β, IL-6, and IL-17, thereby alleviating the inflammatory response in TED (106). (**Figure 5** presents the regulatory mechanism of miRNAs on Inflammatory responses in TED treatment).

### 3.5 The role of miRNAs in other pathological processes

T cells play a critical role in the pathological cascade of TED, directly contributing to tissue inflammation and activating OFs, thereby driving core pathological changes such as adipose proliferation and fibrosis (107). Consequently, therapies targeting specific T-cell subsets or their cytokines have emerged as a key focus in the development of novel TED treatments. The study has shown that miR-183 and miR-96 are significantly upregulated in CD4^+^ T cells from the peripheral blood of patients with TED, and are also highly expressed in activated human and murine T cells *in vitro*. Overexpression of miR-183 and miR-96 inhibits the EGR-1/PTEN/Akt signaling pathway, promotes T-cell proliferation, and thus accelerates TED progression (108). These findings highlight the therapeutic potential of inhibitors targeting miR-183 and miR-96 in TED. Both miR-146a and IL-17 are involved in autoimmune processes in TED. Research has confirmed that serum IL-17 levels increase with disease activity, while miR-146a expression decreases. Both markers show significant correlation with the Clinical Activity Score (CAS) and are inversely correlated with each other, suggesting their utility as potential biomarkers for assessing TED disease activity (109). Intravenous glucocorticoids (GCs) remain the first-line treatment for moderate-to-severe TED. In a prospective longitudinal study of 30 adult patients with active moderate-to-severe TED eligible for GC therapy, ROC analysis indicated that miR-146a could predict GC responsiveness with a positive predictive value of 100%. These results support miR-146a can serve as an objective new marker for GC sensitivity, and can be used to prevent the ineffective use of GC treatment in TED patients (59). (**Table 1** summarizes the therapeutic mechanisms of miRNAs in TED).

**Table 1:** Summary of the mechanism of miRNA in the treatment of TED.

Mechanism of action	MiRNA expression	Object of study	Target sites of action	Specific regulatory role	The development direction of miRNA	References
Regulate the proliferation and activation of OFs	miR-182-5p(-)	TED-OFs	Smad7	CD34 OFs proliferation ↑; CD34 OFs apoptosis ↓	miR-182-5p mimic	(39)
	miR-101-3p(↑)	TED-OFs	PTX3	OFs proliferation ↓	miR-101-3p mimic	(44)
	miR-21(↑)	TED-OFs	PDCD4	OFs proliferation ↑	miR-21 inhibitor	(47)
	miR-103a-3p(↑)	TED-OFs	TGFBR3	OFs proliferation ↑; Vimentin, fibronectin, fascin ↑, Fibrosis ↑	miR-103a-3 pinhibitor	(50)
	miR-146a, miR-155(↑)	TED-OFs	ZNRF3, PTEN	OFs proliferation ↑; Fibrosis ↑	miR-146a/miR-155 inhibitor	(55)
	miR-155(↑), miR-146a(↓)	TED patients	-	OFs proliferation ↑; Inflammatory response ↑; Fibrosis ↑	miR-155 mimic/miR-146a inhibitor	(56)
	miR-144-3p(↑)	TED patients	-	OFs proliferation ↓; Inflammatory response ↑; Fibrosis ↑	miR-144-3p inhibitor	(60)
Regulate Fibrosis	miRNA-5572(↓)	TED-OFs	F2RL2	COL1A1 ↑, Fibrosis ↑	miRNA-5572 mimic	(68)
	miR-146a(-)	TED patients	TGF-β	Fibronectin, collagen Iα and α-smooth muscle actin ↓, Fibrosis ↓	miR-146a mimic	(69)
	miR-320a(↓)	TED mice	PRDX3	Vimentin, fibronectin, retinoic protein/collagen I ↓; Fibrosis ↓; Oxidative stress ↓; Inflammatory response ↓	miR-320a inhibitor	(72)
	miR-376b(↓)	TED patients	-	HAS2, ICAM1 ↓, Fibrosis ↓;Inflammatory response ↓	miR-376b inhibitor	(42)
	miR-21(↑)	TED-OFs	Smad3	Total collagen, collagen type I ↑, Fibrosis ↑; Proliferation ↑; Apoptosis ↓	miR-21 inhibitor	(78)
Regulate adipogenesis	miR-130a(↑)	TED-OFs	-	Lipid accumulation ↑, Orbital adipose tissue ↑	miR-130a inhibitor	(84)
	miR-182(-)	TED patients	TSHR	Adiponectin, leptin, PPAR-γ, AP2 ↓, Adipogenesis ↓	miR-182 mimic	(86)
	miR-27a/miR-27b(-)	TED patients	-	PPARγ, CCAAT, C/EBPα, C/EBPβ ↓, lipofuscin content stained by Oil Red O ↓, Adipogenesis ↓	miR-27a/miR-27b mimic	(88)
	miR-484/miR-192-5p(-)	TED patients	-	Fatty acid metabolism, ECM receptors ↓	miR-27a/miR-27b mimic	(89)
Regulate inflammatory response	miR-146a(↓)	TED-OFs	NUMB	Inflammatory response ↑	miR-146a inhibitor	(96)
	miR-146a(-)	TED-OFs	-	Inflammatory response ↓	miR-146amimic	(97)
	miR-885-3p(↑)	TED patients	-	Inflammatory response ↓; The sensitivity to glucocorticoids ↑	miR-885-3p mimic	(99)
	miR-155(↑)	TED patients	ITK	Inflammatory response ↓	miR-155 mimic	(106)
Regulate other mechanisms	miR-183, miR-96(↑)	TED patients	EGR-1, PTEN, Akt	T cell proliferation ↑	miR-183/miR-96 inhibitor	(108)
	miR-146a(-)	TED patients	-	Clinical activity score ↓	miR-146a mimic	(109)
	miR-146a(-)	TED patients	-	GC sensitivity ↑	miR-146a mimic	(59)

↑, enhance / promote; ↓, reduce / inhibit; -, not specified for expression changes / not mentioned for target sites.

(↑: enhance/promote; ↓: reduce/inhibit; -: not specified for expression changes/not mentioned for target sites).

## 4 Discussion

miRNAs play a certain regulatory role in the occurrence, development and prognosis outcome of TED(GO/TAO). A comprehensive analysis of existing research reveals the following: (1) In terms of the mechanism of action, current studies primarily focus on four core processes—OFs proliferation and activation, fibrosis, adipogenesis, and inflammatory response. Among these, the molecular pathways underlying of proliferation and fibrosis have been systematically investigated, often revealing multi-pathway regulation. In contrast, research on adipogenesis and inflammatory mechanisms tends to concentrate on single pathways or targets. (2) In terms of the research framework, the current body of work spans three levels: clinical correlation analyses using of derived from TED patients, which have established links between miRNAs such as miR-146a and clinical parameters; *in vitro* cellular studies elucidating the specific functions of individual miRNAs like miR-21; preliminary animal experiments (e.g., the TED mouse model) that help bridge mechanistic insights with clinical translation. (3) In terms of miRNA expression, pro-fibrotic/proliferative miRNAs (such as miR-21, miR-155) are generally upregulated, promoting disease progression; while protective miRNAs (such as miR-146a) are significantly downregulated, weakening their inhibitory effect. In adipogenesis, miR-130a and the miR-27 family respectively exert promoting and inhibitory effects. (4) Key Regulatory miRNAs: miR-21 and miR-146a emerge as central players in TED pathogenesis. miR-21 acts as a hub promoting fibrosis and driving disease progression, while miR-146a serves as a protective node—its downregulation is associated with fibrosis, inflammation, and other pathological processes. (5) Functional Diversity of miRNAs: The same miRNA can exert diverse functions across different pathological contexts depending on its expression level, reflecting the complexity of miRNA-mediated regulatory networks in TED. For example: ①Upregulate miR-144-3p to promote fibrosis but downregulate and inhibit proliferation; ②Upregulate miR-103a-3p/miR-21 to promote proliferation and fibrosis. ③Notably, miR-146a and miR-155 exhibit context-dependent functional diversity, sometimes even displaying opposing roles. For example, upregulation of miR-146a promotes OFs proliferation while inhibiting fibrosis and inflammation. In contrast, upregulation of miR-155 enhances both proliferation and fibrosis in OFs, and can either promote or suppress inflammation depending on its specific target genes. We believe that this functional versatility to multiple factors, including cell type specificity, target gene repertoire, expression dynamics, miRNA interactions, and disease stage. These observations underscore that miRNA functions should be interpreted within specific biological contexts rather than being simplistically categorized as pro-inflammatory or anti-inflammatory. Future research should focus on elucidating the intricate regulatory networks of these miRNAs, ultimately providing new targets for precision intervention in inflammatory diseases. (6) Biomarker Potential: miR-885-3p and miR-146a show promise in predicting drug sensitivity of OFs, potentially guiding treatment strategies. Meanwhile, miR-146a and serum miRNAs such as miR-484 and miR-192-5p are associated with disease activity, offering potential non-invasive circulating biomarkers for monitoring TED.

Although current studies have initially depicted the regulatory map of miRNAs in TED, there are still several significant deficiencies: (1) Uneven depth of mechanistic investigation: The roles of miRNAs in adipogenesis and inflammatory responses are still poorly understood in an integrated manner. Most studies remain at the level of phenotypic association, lacking systematic dissection of upstream and downstream signaling pathways. Furthermore, there has been little exploration of how miRNA-mediated networks coordinate cross-talk between different pathological processes. (2) In terms of the research framework, the current body of work spans three levels: clinical correlation analyses using of derived from TED patients, which have established links between miRNAs such as miR-146a and clinical parameters; *in vitro* cellular studies elucidating the specific functions of individual miRNAs like miR-21; preliminary animal experiments (e.g., the TED mouse model) that help bridge mechanistic insights with clinical translation. It is worth noting that the “TED mouse model” currently in use typically refers to an experimental system constructed through specific methods (such as AAV-mediated overexpression of TSHR, immune adjuvant induction, etc.), which can simulate some pathological features of TED (such as orbital inflammation, adipose hyperplasia, etc.), but has not yet formed a fully comprehensive standard model that is widely recognized for human diseases. The existing models mostly only reproduce the local characteristics of the disease, and there are still differences and limitations in their induction strategies, phenotypic stability, and clinical relevance. (3) Fragmented research perspectives: The majority of studies focus on the function of individual miRNAs, overlooking the fact that miRNAs typically operate as part of cooperative or antagonistic regulatory networks *in vivo*. For instance, it remains unknown how pro-fibrotic miRNAs such as miR-21 and anti-fibrotic miRNAs such as miR-146a balance each other in TED, and the hierarchical organization of such networks is still largely unexplored. (4) Weaknesses in translational development: Aside from partial clinical validation of the association between miR-146a and disease activity, most miRNA candidates have not been systematically evaluated in large, multicenter clinical cohorts. Their potential as diagnostic biomarkers or therapeutic targets remains largely unassessed in terms of feasibility, specificity, and safety, significantly limiting the clinical translation of related discoveries.

Therefore, future work should be systematically carried out in the following aspects. Firstly, a multi-level and integrated research framework must be established. Emerging technologies—such as 3D organoid culture and tissue-engineered models—should be employed to investigate OFs behavior within simulated orbital microenvironments. Concurrently, greater emphasis should be placed on *in vivo* functional validation. This includes developing more physiologically relevant TED animal models and performing systematic gain and loss of function studies on key hub miRNAs (e.g., miR-21 and miR-146a) to assess their therapeutic potential and elucidate their systemic effects. Secondly, a shift from a single-molecule approach to a network-based regulatory perspective is essential. Multi-omics technologies (e.g., transcriptomics and proteomics), integrated with bioinformatics analysis, should be utilized to comprehensively map the miRNA–mRNA–protein regulatory network in TED. Research efforts should focus on elucidating the interactions between core miRNAs (e.g., miR-21 and miR-146a) and key signaling pathways to identify central regulatory nodes and potential compensatory mechanisms in disease pathogenesis. Although multi-target regulation of the network (such as combined intervention with multiple miRNAs) holds theoretical promise, its clinical application still faces challenges such as delivery efficiency, off-target risks, and greater complexity in toxicity and feasibility compared to single-target strategies. These issues need to be carefully evaluated in subsequent studies. Finally, efforts should be made to vigorously promote the implementation of clinical transformation. On one hand, through prospective large-scale clinical cohort studies, the reliability of key miRNA combinations (such as miR-146a and miR-21) as markers for diagnosing disease activity, predicting therapeutic efficacy, and judging prognosis needs to be verified. In parallel, targeted miRNA-based therapeutics—such as miR-21 inhibitors or miR-146a mimics—should be explored. However, the clinical application of this approach faces significant obstacles. The key challenge is targeted delivery, which requires nanocarriers to achieve ocular tissue specificity and minimize systemic side effects. Additionally, off-target effects and long-term safety must be evaluated using multi-omics technologies and chronic toxicity studies in animal models. Furthermore, individualized strategies based on patients’ miRNA expression profiles are essential for advancing miRNA therapy toward clinical translation.
